# Gut Microbiota Dysbiosis and Associated Gut Health Outcomes Among Alcohol Consumers in Musanze District of Rwanda: A Cross-Sectional Study

**DOI:** 10.24248/eahrj.v9i1.836

**Published:** 2025-09-30

**Authors:** Christophe Higiro, Thierry Habyarimana, Cedrick Izere, Callixte Yadufashije, Francois Niyongabo Niyonzima

**Affiliations:** a Department of Biomedical Laboratory Sciences, Faculty of AFS, INES-Ruhengeri, Musanze-155, Rwanda; b Department of Public Health, Graduate School, Kampala University, Uganda; c Department of Math, Science, and PE, CE, University of Rwanda, Rwamagana-55, Rwanda.

## Abstract

**Background:**

Excessive consumption of alcohol contributes to gut dysbiosis, leading to adverse gut health outcomes such as gastrointestinal diseases. About 1.6%, or 200,000, Rwandans between 14 and 64 years old abuse alcohol. But there is a paucity of information on the effects of alcohol on intestinal health. This was a cross-sectional study carried out to determine the gut microbial imbalance and associated outcomes among alcohol consumers in the Musanze district.

**Methods:**

A total of 50 participants were recruited, of which 25 were alcohol consumers, while the remaining 25 were control subjects. Stool samples were collected and transported to the INES Ruhengeri clinical microbiology laboratory for microbial identification. Gut outcomes associated with alcohol consumption were evaluated by an interview-administered questionnaire. An independent t-test was performed to test for the microbial mean difference between alcohol consumers and non-alcohol consumers, while a chi-square test was performed to evaluate associations between gut dysbiosis and outcomes.

**Results:**

*Escherichia coli* (17.5%) was the most predominant among alcohol consumers, while *Lactobacillus* (17.3%) was the most predominant among control subjects. There was a significant association between alcohol consumption and gut microbial alteration to *E. coli* (x^2^ = 4.2, *P*=.04), *Enterococcus faecalis* (x^2^ = 9.9, *P*=.00165), *Lactobacillus* sp. (x^2^ = 16.4, *P*=.000051), *Bacillus* sp. (x^2^ = 5.8, *P*=.016), *S. epidermidis* (x^2^ = 11.7, *P* =.000625), *S. pyogenes* (x^2^ = 3.9, *P*=.048), and the overall association was statistically significant (x^2^ = 65.75, *P* <.00001). The association between gut microbiota dysbiosis and gut health outcomes was also significant for *Pseudomonas aeruginosa* (x^2^ = 18.3, *P*=.001078), *S. pyogenes* (x^2^ = 12.1, *P*=.016623), *Enterococcus faecalis* (x^2^ = 11, *P*=.026564) and the overall association (x^2^ = 113.703, *P*<.00001) with the imbalanced microbiota and outcomes being statistically significant. The odd ratio (OR) for pathogenic bacteria to non-pathogenic bacteria was OR = 5.11>1.

**Conclusion:**

Alcohol consumption is associated with gut microbiota dysbiosis, which predisposes to intestinal disorders. Excessive consumption of alcohol should be stopped to prevent devastating outcomes to intestinal health.

## BACKGROUND

Gut microbiota refers to the group of microorganisms, such as archaea, bacteria, viruses, and fungi, within the gastrointestinal tract. The gut harbours a large number of microorganisms, of which only 1,000 species of bacteria are known, and the phyla *Firmicutes* and *Bacteroidetes* are the majority in the gut.^1^ Gut microbiota plays a huge role in the extraction of energy from food, synthesis of amino acids, vitamins, and short-chain fatty acids, and fights against pathogens by guarding the integrity of the intestinal epithelium.^2^

Alcohol consumption has become habitual across societies and history.^3^ For the last 200 years, alcohol consumption has been linked to negative outcomes among consumers.^4^ For instance, a decade ago, researchers in microbiology focusing on the effect of alcohol on the intestinal microbiota, and both its effects and role in the human body were reported.^5,6^ It was observed that individuals with significant microbiota dysbiosis also exhibit depression, a leaking gut, an elevated intestinal permeability, anxiety, move of luminal bio-compounds, and severe diseases-associated with the alcohol.^6^

Excessive alcohol consumption has been associated with gut microbiota changes in the small and large intestines, especially overgrowth of Gram-negative bacteria and bacterial diversity alterations. Aldehydes produced by alcohol metabolism in the gut generate reactive oxygen species that induce pro-inflammatory responses and lead to gut epithelial barrier malfunction. This increases bacterial transcytosis and causes bacterial products, including endotoxins, bacterial deoxyribonucleic acids, and other pathogen-associated molecular patterns, to move from the intestinal tract to the liver, causing liver damage.^7,8^

Worldwide, alcohol intake is ranked the fifth threat of unreasonable death and disability among people between the ages of 15 and 49.^9^ Alcohol and its metabolites are classified as group 1 carcinogens, along with tobacco and asbestos. Although alcohol use is significant, it isn't enough to induce clinically pertinent organ damage. Many other factors, including alcohol, induce gut microbiota dysbiosis and can consequently influence alcohol-related diseases.^10,11^

Alcohol intake can evoke systemic pro-inflammatory alterations via two gastrointestinal-intermediated mechanisms. The first one is altering the gut microbiome composition and/or function, known as dysbiosis, leading to the rise of lipopolysaccharide, and the second is an alteration of intestinal integrity allowing the passage of intraluminal lipopolysaccharide into the systemic circulation. Studies reported that bacterial translocation triggered by alcohol consumption is caused by inflammatory cytokines’ release as a result of immune activation by lipopolysaccharide, a significant element of the outer membrane of Gram-negative bacteria.^12,13^

The consumption of alcohol is linked to a lessening of bacteria belonging to the phylum *Bacillus* and bacteria that secrete butyrate, typically considered anti-inflammatory. On the other hand, *Proteobacteria* are commonly thought to be pro-inflammatory and are increased.^11,14^ As a result, pro-inflammatory triggers are induced, as well as intestinal hyperpermeability, gut leakiness, and endotoxemia, all of which can contribute to inflammatory bowel disorders, irritable bowel syndrome, and food allergies.^15,16^

Alcohol consumption has increased in the 21^st^ century in different countries of the world, including Rwanda. This study was carried out to provide awareness of alcohol's effect on intestinal health. Related studies were carried out in many different countries,^11,13^ but none was carried out in Rwanda. Therefore, this study was carried out in Rwanda, specifically in Musanze district, to provide knowledge about how alcohol consumption induces gut microbiota dysbiosis, which can lead to loss of homeostasis and other health complications.

## METHODS

### Area of Investigation

This study was carried out in the Musanze district, Northern Province, in the Muhoza sector. Musanze is the country's fourth-largest town and is rapidly developing into a vibrant metropolis. It is one of Rwanda's thirty districts and one of the five districts in the Northern Province. Muhoza sector is one of 15 sectors of Musanze district, where Ruhengeri airport is located. The Muhoza sector office is situated near to Musanze YEGO centre and close to Mobisol. Musanze district was chosen because it is a growing tourist city gathering people from different backgrounds, and this may favour substance abuse, including excessive consumption of alcohol.

### Study Design

This was a cross-sectional study carried out from September 2021 to January 2022. A questionnaire was utilised to gather information related to the gut health outcomes induced by gut microbiota dysbiosis. The pain in the lower part of the abdomen, bloating and gas, diarrhoea, changes in bowel habits, satiety, and loss of appetite were the outcomes of interest considered in the study.

### Sampling

Snowball sampling was used to recruit alcohol consumers, while purposive sampling was used to recruit non-alcohol consumers in a community setting.

### Study Population and Sample Size

Twenty-five chronic alcohol consumers were recruited as cases, and 25 non-alcohol consumers as a control group.

### Inclusion Criteria

Alcohol and non-alcohol consumers with chronic conditions who voluntarily accepted to participate in the study were recruited.

### Exclusion Criteria

Alcohol consumers with GIT treatment were excluded from the study. Participants with gastric outcomes such as cancer and other diseases were also excluded. An interview-administered questionnaire was used to collect information on the gut health outcomes induced by gut microbiota dysbiosis. Consequently, these questionnaires facilitated the inclusion or exclusion of participants based on their responses.

### Collection of Stool Samples

Stool samples were collected and put in sterile Stuart plastic containers to avoid contamination. Stool samples were transported to the INES Ruhengeri microbiology laboratory for microbial and biochemical analysis.

### Macroscopic Examination of Stool Samples

The macroscopic examination involved the observation of stool specimens with the naked eye. Identified abnormalities were based on colour and consistency, as well as the presence or absence of blood and mucus.

### Culture Media Preparation

MacConkey agar (MCA), mannitol salt agar (MSA), and blood agar (BA) were used as culture media on which faecal samples were cultured. Following the manufacturer's instructions, grams of each of the culture media were separately dissolved in corresponding millilitres of distilled water. This was followed by heating with repeated gentle agitation for 2 min to allow a complete dissolution. The culture media were then autoclaved for 15 min at 15 psi and 121°C. Finally, they were cooled at 45°C and poured into different Petri dishes for solidification.

### Inoculation, Incubation, and Gram Staining

The streak method was used to inoculate specimens onto Petri dishes containing blood agar, MCA, and MSA. The cultural Petri plates were grown overnight at 37°C. Growth was observed in terms of bacterial colony formation. Identified colonies were separately smeared and fixed on different slides, and finally the Gram staining technique was performed. After air drying, the stained slides were observed under a microscope at 100× objective.

### Biochemical Tests

Kligler iron agar test (KIA) was used to identify enterobacteria based on the double fermentation of sugar and the production of hydrogen sulphide. The Simmon's citrate agar test (SCA) was performed to differentiate members of enterobacteria capable of using citrate as a carbon source. Urea broth was utilised to find out the microorganisms that fractionate urea by the production of urease. Sulphide-indole-motility (SIM) was utilised to decide which bacteria possess the capacity to reduce SO_4_^2−^, to secrete indole, and to exhibit motility. To assess for indole secretion, three drops of Kovac's reagent were supplemented. The appearance of a pink ring showed indole production. The catalase slide method was applied to distinguish *Staphylococcus* and *Streptococcus* species. The coagulase method was utilised to confirm *Staphylococcus aureus*. The oxidase test was conducted using an oxidase reagent. The appearance of a blue colour within 15 s was an indication of positive results.

### Antibiotic Susceptibility Test

To confirm *Staphylococcus epidermidis* and *Streptococcus pyogenes*, the antibiogram was performed to examine the sensitivity or resistance of the suspected bacteria to novobiocin and bacitracin, respectively. The disc agar diffusion procedure was utilised. Test bacteria grown on blood agar plates at 37°C were dissolved in 0.85% (w/v) NaCl and set to 0.5 McFarland standard turbidity. Using a standardised suspension, a cotton swab was utilised to inoculate the Mueller-Hinton agar plate. The plates were left for 30 min. The discs were transferred directly to the sensitivity plates using sterile forceps. Within 30 min of application, the plates were turned inside out and incubated overnight at 37°C to examine the inhibition zone around the plates.

### Statistical Analysis

SPSS version 22 was used to analyse data. An independent t-test was used to test for mean differences in intestinal microbial composition among alcohol consumers and non-consumers. The association between alcohol consumption and dysbiosis of the gut microbiota, and the effect of dysbiosis of the gut microbiota on gut health in alcohol consumers, were analysed using the chi-square. The odds ratio was used to analyse the ratio of pathogenic bacteria to non-pathogenic bacteria. The significance was considered when P < .05 for the T-test and Chi-square, while for the odd ratio OR > 1.

### Ethical Consideration

The ethical clearance was sought from the INES-Ruhengeri research committee. Written informed consent was signed by each participant before stool sample collection. Participants were given unique identification numbers to protect their identity.

## RESULTS

### Demographic Characteristics of the Participants

In the present study, alcohol drinker participants were categorised into four age range groups. Most drinkers were males in the 19–23- and 24–28-year-range groups (Table 1).

**TABLE 1:** Age and Gender of Participants (N = 50)

Demographics	Alcohol consumer		Control subjects	
	Frequency	(%)	Frequency	(%)
Age (years)				
19–23	8	32	10	40
24–28	10	40	9	36
29–33	5	20	2	8
34–38	2	8	4	16
Total	25	100	25	100
Gender				
Male	20	80	7	28
Female	5	20	18	72
Total	25	100	25	100

### Profiles of Gut Microbial Composition among Alcohol Consumers and Non-Consumers

**FIGURE 1:**
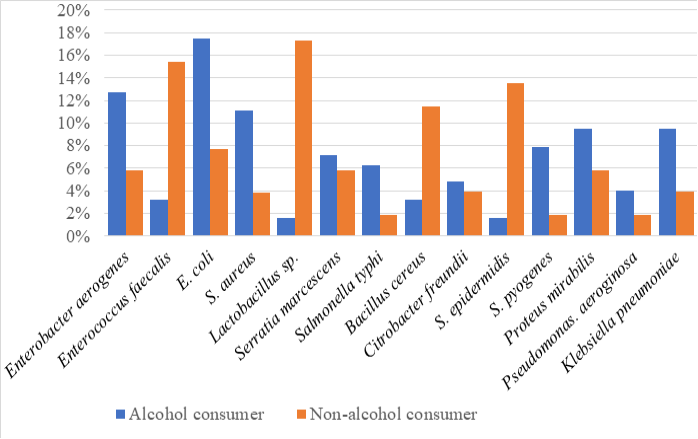
Profiles of Gut Microbial Composition Among Alcohol Consumers and Non-Consumers

The proportions of gut microbial composition isolated from stool samples of 25 alcoholic and 25 non-alcoholic users, respectively (Table 2), were *Enterobacter aerogenes* (12.7%, 5.8%), *Enterococcus faecalis* (3.2%, 15.4%), *Staphylococcus aureus* (11.1%, 3.8%), *E. coli* (17.5%, 7.7%), *Lactobacillus* sp. (1.6%, 17.3%), *Serratia marcescens* (7.14%, 5.8%), *Salmonella typhi* (6.3%, 2%), *Bacillus cereus* (3.2%, 11.5%), *Citrobacter freundii* (4.8%, 3.9%), *Staphylococcus epidermidis* (1.6%, 13.5%), *Streptococcus pyogenes* (7.9%, 2%), *Proteus mirabilis* (9.5%, 5.8%), *Pseudomonas aeruginosa* (4%, 2%) and *Klebsiella pneumoniae* (9.5%, 3.9%) (Figure 1).

**TABLE 2:** Comparison of Gut Microbiota Between Alcohol Consumers and Non-Alcohol Consumers

Bacteria	Alcohol consumer	Non-alcohol consumer	Total	D	MD	(D-MD)2	SD	SDE	T-test	*P-value*
*Enterobacter aerogenes*	16	6	22	10		70.6				
*Bacillus cereus*	4	12	16	−8		92.2				
*Citrobacter freundii*	6	4	10	2		0.2				
*E. coli*	22	8	30	14		153.8				
*Enterococcus faecalis*	4	16	20	−12		185				
*Klebsiella pneumoniae*	12	4	16	8		41				
*Lactobacillus* sp.	2	18	20	−16		309.8				
*Proteus mirabilis*	12	6	18	6		19.4				
*Pseudomonas. Aeruginosa*	5	2	7	3		2				
*S. aureus*	14	4	18	10		70.6				
*S. epidermidis*	2	14	16	−12		185				
*S. pyogenes*	10	2	12	8		41				
*Salmonella typhi*	8	2	10	6		19.4				
*Serratia marcescens*	9	6	15	3		2				
Total	126	104	230	22	1.6	1192	9.6	2.6	0.6	0.5

D: Difference, MD: Mean Difference, SD: Standard Deviation, SDE: Standard

### Association Between Gut Microbial Imbalance and Alcohol Consumption

An overall statistically significant association between gut microbial community dysbiosis and alcohol consumption was observed. It was significantly shown by *Enterococcus faecalis, Escherichia coli, Staphylococcus aureus, Lactobacilli* sp., *Bacillus* sp, *Staphylococcus epidermidis*, and *Streptococcus pyogenes*. There was no significant association observed for other bacteria. Table 3 presents the df, chi-square, and *p*-values considering the association of gut microbiota dysbiosis and alcohol consumption.

**TABLE 3:** Association of Gut Microbial Dysbiosis and Alcohol Consumption

Bacteria	Alcohol consumer		Non-alcohol consumer		Total	Df	X2	*P-value*
	O	E	O	E				
*Enterobacter aerogenes*	16	12.1	6	9.9	22	1	2.8	.094264
*Bacillus cereus*	4	8.8	12	7.2	16	1	5.8	.016026
*Citrobacter freundii*	6	5.5	4	4.5	10	1	0.15	.698535
*E. coli*	22	16.4	8	13.6	30	1	4.2	.040424
*Enterococcus faecalis*	4	11	16	9	20	1	9.9	.001653
*Klebsiella pneumoniae*	12	8.8	4	7.2	16	1	2.6	.106864
*Lactobacillus* sp.	2	11	18	9	20	1	16.4	.000051
*Proteus mirabilis*	12	9.9	6	8.1	18	1	0.9	.342782
*Pseudomonas aeruginosa*	5	3.8	2	3.2	7	1	0.9	.342782
*S. aureus*	14	9.9	4	8.1	18	1	3.8	.051253
*S. epidermidis*	2	8.8	14	7.2	16	1	11.7	.000625
*S. pyogenes*	10	6.6	2	5.4	12	1	3.9	.048286
*Salmonella typhi*	8	5.5	2	4.5	10	1	2.5	.113846
*Serratia marcescens*	9	8.2	6	6.8	15	1	0.2	.654721
Total	126		104		230	13	65.75	< .00001

Df: Degree of Freedom, O: Observed Value, x2: Chi Square, E: Expected Value

### Association Between Gut Microbial Dysbiosis and Gut Health Outcomes

The association between gut microbial dysbiosis and gut health outcomes among alcohol consumers was determined to be statistically significant (x^2^=113.703, *P*<.00001). A summary of the statistical association between gut microbial imbalance and the indicated gut health outcomes is illustrated in Table 4.

**TABLE 4:** Association Between Gut Microbial Dysbiosis and Gut Health Outcomes

Gut health outcomes	Pain in the lower part of the abdomen	Bloating & gas	Diarrhoea	Changes in bowel habit	Satiety & loss of appetite	Total	Df	X2	*P-value*
Bacteria									
*Enterobacter aerogenes*	8 (11)	12 (10.6)	10 (10.2)	6 (9.4)	16 (10.5)	52	4	4.903	.297396
*Bacillus cereus*	0 (2.1)	4 (2)	4 (1.9)	2 (1.8)	0 (2) 10	4	8.42	.07735	
*Citrobacter freundii*	2 (2.7)	2 (2.6)	0 (2.5)	6 (2.3)	3 (2.6)	13	4	8.66	.070183
*E. coli*	14 (12.3)	10 (11.9)	16 (11.4)	12 (10.5)	6 (11.7)	58	4	5.2	.267385
*Enterococcus faecalis*	0 (1.2)	4 (1.2)	0 (1.1)	2 (1)	0 (1.2)	6	4	11	.026564
*Klebsiella pneumoniae*	8 (10.2)	4 (9.8)	14 (9.4)	8 (8.7)	14 (9.7)	48	4	7.95	.093427
*Lactobacillus* sp.	2 (1.2)	0 (1.2)	2 (1.1)	0 (1)	2 (1.2)	6	4	3.9	.419709
*Proteus mirabilis*	14 (9.8)	10 (9.4)	6 (9)	6 (8.3)	10 (9.3)	46	4	3.48	.480926
*Pseudomonas. aeruginosa*	0 (1.7)	2 (1.6)	6 (1.5)	0 (1.4)	0 (1.6)	8	4	18.3	.001078
*S. aureus*	10 (9.1)	12 (8.8)	8 (8.4)	2 (7.7)	11 (8.7)	43	4	5.99	.199896
*S. epidermidis*	0 (0.8)	2 (0.8)	0 (0.7)	2 (0.7)	0 (0.8)	4	4	6.5	.16479
*S. pyogenes*	12 (6.1)	5 (5.9)	2 (5.7)	8 (5.2)	2 (5.8)	29	4	12.1	.016623
*Salmonella typhi*	8 (5.1)	2 (4.9)	4 (4.7)	8 (4.3)	2 (4.8)	24	4	8.1	.087983
*Serratia marcescens*	2 (5.9)	8 (5.7)	2 (5.5)	6 (5)	10 (5.6)	28	4	9.2	.05629
Total	80 (79.2)	77 (76.4)	74 (73.1)	68 (67.3)	76 (75.5))	375 (371.5)	52	113.703	< .00001

### Ratio Of Pathogenic Bacteria Compared to Non-Pathogenic Bacteria Among Alcohol Consumers

In the present assessment, the ratio of pathogenic bacteria to non-pathogenic bacteria was checked among alcohol consumers. The odds ratio (OR) and risk ratio (RR) noticed were 5.11 and 2.1, respectively. Table 5 shows the odds ratio and risk ratio of pathogenic bacteria to non-pathogenic bacteria in alcohol users.

**TABLE 5:** Ratio of Pathogenic Bacteria to Non-Pathogenic Bacteria

	Alcohol consumer		Non-alcohol consumer		Total
	P.M	N-P.M	P.M	N-P.M	
*Enterobacter aerogenes*	16	0	6	0	22
*Bacillus cereus*	0	4	0	12	16
*Citrobacter freundii*	6	0	4	0	10
*E. coli*	0	22	0	8	30
*Enterococcus faecalis*	0	4	0	16	20
*Klebsiella pneumoniae*	12	0	4	0	16
*Lactobacillus* sp.	0	2	0	18	20
*Proteus mirabilis*	12	0	6	0	18
*Pseudomonas. aeruginosa*	5	0	2	0	7
*S. aureus*	14	0	4	0	18
*S. epidermidis*	0	2	0	14	16
*S. pyogenes*	10	0	2	0	12
*Salmonella typhi*	8	0	2	0	10
*Serratia marcescens*	9	0	6	0	15
Total	92	34	36	68	230
Risk in cases	0.7				
Risk in control			0.34		

OR=5.11; R=2.1

## DISCUSSION

Alcohol consumption is common and incorporated in different cultures, which favours males drinking more often and heavily than women.^17^ Among 25 alcohol consumers recruited, the dominant age range was 24–28, followed by 19 to 23. The age range of 19–23 was predominant, followed by 24–28 among control subjects or non-alcohol consumers. Males (80%) were the dominant gender among alcohol consumers, while among control subjects, females (72%) were the dominant gender. Worldwide, 26.5% of all 15- and 19-year-olds are currently alcohol consumers, while at the ages of 20 and 24, they engage in heavy episodic drinking, particularly males with a high prevalence.^18^ Kanyoni and others,^19^ also reported that 7.6% of Rwandans aged under 35 years are either addicted or abuse alcohol. In Rwanda, for instance, it is common for adult males to use alcohol for relaxation and fun. Also, alcohol drinking is used as cultural symbol of manliness/masculinity, which explains the high prevalence of alcohol drinking among men in this study.

Chronic and heavy alcohol intakes induce changes in bacteria in the small and large intestines, especially Gram-negative bacteria, and alterations in bacterial diversity in alcohol users compared to non-alcohol users.^8^ Alcohol consumers present a higher proportion of pathogenic bacteria, mostly Gram-negative bacteria. This is due to the fact that ethanol has been found to have a great negative impact on Gram-positive bacteria, including *Lactobacilli* that contribute a lot to the gut microbiota eubiosis.^20^ In various microorganisms such as *E. coli*, an imbalance in the intestinal microbial community was observed between the two groups, 2.75 times higher in alcohol users compared to the control group. *Enterobacter aerogenes* was 2.6 times higher among alcohol consumers. *Enterococcus faecalis* and *Lactobacilli* sp. were more common in non-alcoholic users compared to alcohol users. Current results are consistent with studies conducted in patients with chronic alcohol abuse, where bacteria from the phylum *Proteobacteria*, mainly *Enterobacteriaceae*, were observed in abundance.^21^

In humans, continuous consumption of alcohol has been shown to be associated not only with the overgrowth of bacteria in the small intestine but also with changes in the makeup of mucosal-related microbial flora in sigmoid biopsies.^11,22^ Findings of this study revealed that there was an overall statistically significant association between gut microbial community dysbiosis and alcohol consumption. This association was significantly indicated by *Enterococcus faecalis, Escherichia coli, Staphylococcus aureus, Lactobacilli* sp., *Bacillus* sp., *Staphylococcus epidermidis*, and *Streptococcus pyogenes*. Previous studies observed the low levels of *Bacteroides* and *Lactobacillus* species in alcoholism and the high abundance of *Proteobacteria* and *Fusobacteria*.^11,23^

A similar study reported that alcohol abuse promoted overgrowth of gut microbiota in both preclinical and human models, primarily in the upper small intestines.^24^ Heavy drinkers present with low bacteria from the phylum *Bacteroidetes* and butyric acid-producing bacteria, which are generally considered to be anti-inflammatory, and an abundance of bacteria from the phylum *Proteobacteria*, including *Escherichia coli*, *Salmonella, Klebsiella*, *Enterobacter*, and *Shigella*, widely believed to be pro-inflammatory.^14^ Mutlu and others,^11^ reported in research conducted in mice that alcohol intake was correlated with an imbalance in the bacterial family, showing a decrease of beneficial bacteria (*Lactobacillus* sp*., Enterococcus* sp*., Bacillus*, and other beneficial *Firmicutes*), with a converse abundance in the incidence of *Bacteroidetes* and *Verrucomicrobia*.

For this study, the analysis of the association between gut microbiota dysbiosis and gut health outcomes among alcohol consumers was statistically significant (x^2^=113.703, *P*<.00001). Like in the small intestine, alcohol in the colon reduces colonic impeding motility but increases its propulsive motility. Mezey^25^ reported that alcohol consumption significantly decreased the frequency and intensity of partial rectal muscle contractions in healthy individuals. These outcomes can reduce passage time and, therefore, compression of intestinal contents, thus leading to diarrhoea, which often occurs in alcoholism. In addition, drinks with high alcohol levels above 15% appear to impede gastric motility and slow down gastric emptying. As a result, the stomach takes longer to pass, bacterial breakdown of food begins, and the resulting gas can lead to satiety and abdominal discomfort. Alcohol reduces muscle movement in the small intestine, which normally helps hold food for further digestion. These effects can contribute to increased susceptibility to hyperglycaemic foods, shorter transit times, and diarrhoea commonly seen in alcoholism.^26^

The imbalance between beneficial and pathogenic bacteria observed in alcohol consumers leads to decreased gastrointestinal motility and increased intestinal toxins such as lipopolysaccharide A. It later changes the usual functioning of the gastrointestinal tract by inhibiting the transport of glucose and amino acids. Vitamins affect thiamine and vitamin B12, as well as minerals such as calcium and magnesium. Therefore, malabsorption and diarrhoea, flatulence and distension, lower abdominal pain, changes in bowel habits, feeling of fullness, and loss of appetite.^27^

Increased or overgrowth of *Proteobacteria*, usually *Enterobacteriaceae*, with loss of microbial diversity and abundance reported in alcohol consumers, is among the potentially significant factors associated with gastrointestinal disorders. It has been suggested.^28^ For the odd ratio in the present research, the OR was 5.11 for pathogenic bacteria to non-pathogenic bacteria in alcohol users. Because these relevance measures are greater than 1, it implies that consuming alcohol contributed to gut health outcomes. Kuprys and others^29^ also reported a high ratio of intestinal *Enterobacteriaceae* to *Lactobacillus*, which may contribute to intestinal disorders as well as liver damage.

## CONCLUSION

The present study investigated gut microbial dysbiosis and associated gut health outcomes among alcohol consumers. The imbalance of gut microbiota was associated with alcohol consumption, whereby mainly bacteria of the *Enterobacteriaceae* family outgrew other bacteria isolated from the gut among alcohol consumers.

Various gut outcomes, including diarrhoea, pain in the lower part of the abdomen, bloating and gas, change in bowel habits, and loss of appetite, were found to be associated with microbiota dysbiosis. The odds ratio revealed that gut microbiota dysbiosis contributed to the gut health outcomes studied. Deleterious effects of alcohol on gut microbiota are not only localised in the gut; they can reach other vital organs, which later affect the whole-body system. Owing to the increase in alcohol consumption among youth, young people need to be sensitised about the effect of alcohol on gut health. Secondly, the government of Rwanda should reinforce youth education programmes about the effects of alcohol. Thirdly, further research is needed to provide enough information on the restoration of the lost gut microbiota homeostasis induced by alcohol.

### Study limitations

The study patients in the present research were from the Musanze district, one of 30 districts of Rwanda. Thus, the results of this study cannot be generalised to the Rwandan population. Given the sensitive nature of alcohol abuse, it was also difficult to get samples from participants. In addition, we were limited by the lack of molecular techniques to study the microbiome of isolated microorganisms. Hence, the gut microbial imbalance was analysed based on isolated bacteria.
